# Supplementary material for: Efficacy profile of the CYD-TDV dengue vaccine revealed by Bayesian survival analysis of individual-level phase III data
Source: eLife. 2021 Jul 2;10:e65131. doi: 10.7554/eLife.65131 (PMC8321579; doi:10.7554/eLife.65131)
Supplement: Supplementary file 1. [file elife-65131-supp1.docx]

**Supplementary file 1.** Model glossary / parameter table.

| **Symbol^†^** | **Description** | **Comments** |
| --- | --- | --- |
| *a* | Trial arm | Coded 0 = control arm; 1 = vaccine arm |
| *b* | Baseline serostatus | Coded 0 = seronegative; 1 = seropositive |
| *c* | Country |  |
| *d* | Dengue serotype |  |
| *D* | Disease/case type | Coded 0 = active phase; 1 = passive phase by default  Coded 0 = non-hospitalised; 1 = hospitalised if considering hospitalisation  Coded 0 = non-severe; 1 = severe if considering severe disease |
| *α* | Age | Age group 1 = 2-5 years; Age group 2 = 6-11 years; Age group 3 = 12-16 years |
| *t* | Calendar time | Units in days |
| *t** | Time post dose | Units in days |
| *t_F_* | Time of most recent dose |  |
| *λ_abcdD_(t, α)* | Hazard in trial arm *a* for baseline serostatus *b* in country *c* from serotype *d* of disease type *D* at calendar time *t* at age *α* |  |
| *λ_c_(t)* | Baseline hazard in country *c* at time *t* | Assumed to be constant over interval *T_I_ =* 1 day. Each country modelled as quadratic spline where knots *κ_ck_* are fitted parameters |
| ***M_b_*** | Multiplier of baseline hazard for serostatus *b* | Reflects seropositives reduced susceptibility to infection (not disease) due to their previous infection with and immunity to at least one serotype. Fitted with prior *Unif*(0,1). Fixed at 1 for seronegatives. |
| *ρ_cd_* | Proportion of serotype *d* in country *c* | Assumed constant over observation period |
| ***q_cy_*** | Parameters governing serotype proportions *ρ_cd_* in each country *c* (see Methods) | Fitted with prior *Unif*(0,1) |
| ***Z(α)*** | Age specific multiplier of baseline hazard | Piecewise constant step function for age groups 1 (2-5yrs), 2 (6-11yrs) and 3 (12-16 yrs). Value for age group 1 fixed at 1 (baseline). Constant values are fitted parameters with prior *Unif*(0,5). |
| ***K_i,D_*** | Relative risk of disease of type *D* given *i* previous infections. *K_1,0_* := 1 taken as baseline. Risks from tertiary and quaternary infections assumed to be equal, i.e. *K_2D_ = K_3D_*. | *K_0,0_* and *K_2,0_* fitted with prior *Unif*(0,1). *K_1,1_* fitted with prior *Unif*(0,0.5). We fix the ratios *K_0,1_ / K_1,1_* = *K_2,1_ / K_1,1_* = 0.25 (as in [10]) |
| *φ_cD_(α)* | Seropositive disease risk where number of previous infections unknown | Weighted average of seropositive (monotypic and multitypic) relative risks of disease type *D* given participants age *α* and country *c* |
| *R_abcD_(α)* | Relative risk of disease of type *D* in stratum *a, b, c.* | Term wrapper of: i) seronegative (*K_0,D_*) and seropositive (*φ_cD_(α)*) disease risk; ii) change in long term disease risk from “silent infection” vaccination |
| *δ_a,Vac_* | Kronecker delta | 0 if subject in control arm, 1 if subject in vaccine arm |
| ***τ_b_*** | Mean duration of transient immunity for serostatus *b* | Fitted with prior *Unif*(0,10) for *b = 0* (seronegatives) and *Unif*(0,20) for *b = 1* (seropositives) |
| *I_bd_(α)* | Initial transient immunity for baseline serostatus *b*, serotype *d* at age *α* | Sum of *A_b_(α)* and *s_bd_* below. Prior is *Unif*(-10,1) for *b = 0* (seronegatives) and *Unif*(0,1) for *b = 1* (seropositives) |
| ***A_b_(α)*** | Transient immunity with age for serostatus *b* | Piecewise constant step function for age groups 1 (2-5yrs), 2 (6-11yrs) and 3 (12-16 yrs). Constant values are fitted parameters with prior *Unif*(-10,1) for *b = 0* (seronegatives) and *Unif*(0,1) for *b = 1* (seropositives) |
| ***s_bd_*** | Intercept of transient immunity for baseline serostatus *b* and serotype *d* | Fixed at 0 for serotype *d* = 1, otherwise fitted with prior *Unif*(-10,1) for *b = 0* (seronegatives) and *Unif*(0,1) for *b = 1* (seropositives) |
| *I*_bd_(α,t,t_F_)* | transient immunity remaining at time *t* after most recent dose *t_F_* against serotype *d* for baseline serostatus *b* for age *α* |  |
| ***h_c_*** | Historical hazard of infection in country *c.* | Fitted with prior *Unif*(0,1) |
| ***κ_ck_*** | *k*’th knot of spline in country *c* | Knots spaced at 1/3-year (approx. 4-month) intervals between 3^rd^ Oct 2011 and 31^st^ Jan 2015 . Knots explicitly determine values of *λ_c_(t)*. Logged knots are fitted parameters with prior *Unif*(-6,0) |
| *β_ick_* | Coefficient of *i^th^* power of *k^th^* polynomial of baseline hazard spline in country *c* |  |
| *p_0c_(α), p_1c_(α)* | Probabilities of exactly 0 and 1 prior infections by age *α* in country *c* |  |
| *λ_abcD_(t, α)* | Hazard from any serotype | Sum of *λ_abcdD_(t, α)* over all serotypes *d* |
| *λ*_abc_(t, α)* | Hazard from any serotype in either trial phase | Wrapper of *λ_abcD_(t, α)* |
| *Λ_abc_(t_S_,t_E_,α)* | Integrated hazard between *t_S_* and *t_E_* |  |
|  | Probability of remaining disease-free between *t_S_* and *t_E_* |  |
|  | Probability or remaining disease-free between *t_S_* and *t_E_* and contracting disease of type *D* from serotype *d* at time *t_F_* |  |
|  | Probability of clinical outcome *C.* |  |
| *π_bc_(α)* | Probability of serostatus *b* in country *c* at age *α* |  |
| *L(θ)* | Likelihood of parameter set *θ* |  |
| *HR_S_(t*)* | Hazard ratio (vaccine:control) in stratum *S* due to any serotype or disease/case type after *t** days post first dose |  |
| *HR_S_(t*,d,D)* | Hazard ratio (vaccine:control) of disease type *D* in stratum *S* due to serotype *d* after *t** days post first dose |  |
|  | Probability of remaining disease-free *t** days post first dose, aggregated across stratum *S* |  |
| *AR(Trial Period)* | Attack rate over trial period (either active phase or passive phase) |  |

^†^ Symbols in bold font refer to fitted parameters.
